# Clip opening while locked after transcatheter edge-to-edge mitral valve repair with different onset times: a case series

**DOI:** 10.1093/ehjcr/ytae322

**Published:** 2024-07-12

**Authors:** Hiroki Matsuzoe, Kazuki Mizutani, Naoko Soejima Onishi, Ayano Yoshida, Takayuki Kawamura, Masafumi Ueno, Genichi Sakaguchi, Gaku Nakazawa

**Affiliations:** Division of Cardiology, Kindai University Faculty of Medicine, 377-2 Onohigashi, Osakasayama City, Osaka 589-8511, Japan; Division of Cardiology, Kindai University Faculty of Medicine, 377-2 Onohigashi, Osakasayama City, Osaka 589-8511, Japan; Division of Cardiology, Kindai University Faculty of Medicine, 377-2 Onohigashi, Osakasayama City, Osaka 589-8511, Japan; Division of Cardiology, Kindai University Faculty of Medicine, 377-2 Onohigashi, Osakasayama City, Osaka 589-8511, Japan; Division of Cardiology, Kindai University Faculty of Medicine, 377-2 Onohigashi, Osakasayama City, Osaka 589-8511, Japan; Division of Cardiology, Kindai University Faculty of Medicine, 377-2 Onohigashi, Osakasayama City, Osaka 589-8511, Japan; Division of Cardiovascular Surgery, Kindai University Faculty of Medicine, Osaka 589-8511, Japan; Division of Cardiology, Kindai University Faculty of Medicine, 377-2 Onohigashi, Osakasayama City, Osaka 589-8511, Japan

**Keywords:** Mitral regurgitation, Transcatheter edge-to-edge mitral valve repair, Clip opening while locked (COWL), Case series

## Abstract

**Background:**

Transcatheter edge-to-edge mitral valve repair is now available in many countries and has achieved favourable therapeutic outcomes. However, there have been no reported cases of clip opening while locked (COWL) during the acute phase using the MitraClip G4 system (Abbott, Abbott Park, IL, USA).

**Case summary:**

We present two cases of COWL occurring at different phases: one immediately after clip release and the other 2 days post-procedure. In both cases, the initial treatment involved the use of the XTW system. Subsequently, an additional XT system was deployed for the deterioration of mitral regurgitation caused by COWL, without any complications.

**Discussion:**

The MitraClip G4 system offers four size variations, providing a larger grasping area and increased flexibility for accessing complex lesions. Furthermore, the complication rate decreased with increasing operator experience and device generation. However, it has been reported that COWL can occur after the clip is deployed during TEER. Although the mechanism of COWL is unclear, the nature and mobility of the valve leaflets and the product specificity of the MitraClip may be involved.

**Learning points**

- Clip opening while locked (COWL) is a phenomenon that describes when clip arm angle increases post-clip deployment during transcatheter edge-to-edge mitral valve repair (TEER).
- Although infrequent, COWL generally occurs after clip release but can also be observed in the subacute phase after TEER, worsening mitral regurgitation grade.
- The diagnosis of COWL should be made using both rotational fluoroscopies to find the angle at which the clip appears most enlarged and transoesophageal echocardiography.

## Introduction

Transcatheter edge-to-edge mitral valve repair (TEER) with MitraClip (Abbott, Abbott Park, IL, USA) is a valid therapeutic option for patients with heart failure and mitral regurgitation (MR).^1^ The G4 system is now available in many countries, and favourable therapeutic effects have been reported. However, the so-called clip opening while locked (COWL) phenomenon, which could lead to MR worsening after TEER, has been reported with an incidence rate of 0.28–0.5%, despite appropriate clip placement with sufficient decrease in MR (urgent medical device correction report from Abbott; released on 8 September 2022, https://www.cardiovascular.abbott/content/dam/cv/cardiovascular/pdf/reports/MitraClip-Urgent-Medical-Device-Correction-September-2022.pdf). Although, reportedly, COWL rarely occurs, when it does manifest, it typically follows the release of the clip. Furthermore, COWL may be observed during the subacute phase following TEER, potentially exacerbating MR severity. Herein, we report the clinical course of two cases of COWL that occurred at different phases: immediately after clip release and 2 days after the procedure.

## Summary figure

**Table:** 

Patient 1	
Date	Events
X-13 years	Pacemaker implantation was performed for sick sinus syndrome.
X-4 months	Shortness of breath [New York Heart Association (NHYA) class III] was also observed.
X-1 month	Despite the guideline-directed medical therapy (GDMT) treatment, chest radiography revealed cardiomegaly, and transthoracic echocardiography (TTE) showed severe MR due to a flat valve and severe tricuspid regurgitation with non-coapting valve leaflets.
X-2 days	Transoesophageal echocardiogram (TEE) confirmed inadequate coaptation of the mitral leaflets leading to severe MR.
Day 1	Admission to our institution to undergo TEER with MitraClip.
Day 3	Transcatheter edge-to-edge mitral valve repair with MitraClip was performed. Immediately after the first clip was placed, immediate MR deterioration and COWL were confirmed by closer observation. An additional clip was inserted, and the MR was reduced.
Day 7	Symptoms were improved on additional GDMT, and the patient was discharged from our institution.
Patient 2	
Date	Events
X-2 years	Surgical repair by total aortic arch replacement (TAR) for acute Stanford type A aortic dissection.
X-2 months	Hospitalization at other hospitals for decompensated heart failure (NHYA class III). Transthoracic echocardiography revealed severe MR due to mitral valve prolapse (MVP), and the patient was discharged 14 days after receiving optimal diuretic therapy.
X-1 month	Referred to our outpatient department for further treatment.
X-3 days	Transoesophageal echocardiogram confirming severe MR due to flailing of the posterior mitral leaflet (PML).
Day 1	Admitted to our institution to undergo TEER with MitraClip.
Day 4	Transcatheter edge-to-edge mitral valve repair with MitraClip was performed.
Day 6	Systolic regurgitation was audible and suspected deterioration of MR.
Day 7	Transoesophageal echocardiogram revealed worsening of MR due to the COWL.
Day 8	Additional TEER with MitraClip.
Day 13	Discharged from our institution after adjustment of oral medication.

## Case summary

### Patient 1

An 85-year-old man presenting with worsening shortness of breath was referred to our institution for further investigation of decompensated heart failure and severe MR. He had a previous history of permanent pacemaker implantation for sick sinus syndrome 13 years ago. Despite the administration of appropriate oral medications for heart failure, significant improvement in MR and symptoms was not observed.

On admission, blood pressure and pulse rate were 115/69 mmHg and 84 b.p.m., respectively. Physical examination revealed a Levine III/VI degree pansystolic murmur at apex and pitting leg oedema. His NYHA class was II.

**Figure 1:**
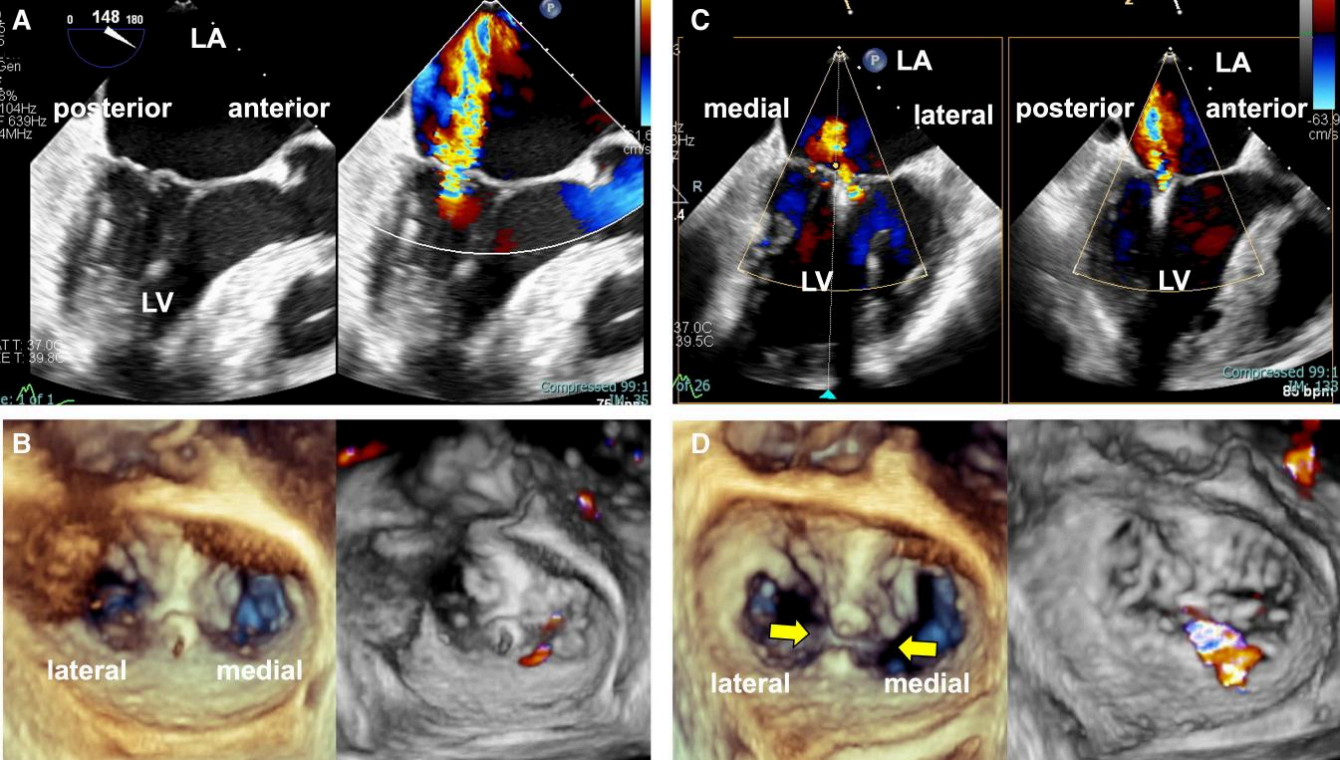
Intra-procedural transoesophageal echocardiogram images of the first transcatheter edge-to-edge mitral valve repair of Patient 1. Preprocedural transoesophageal echocardiogram showing severe mitral regurgitation (*A*). After XTW system deployment, transoesophageal echocardiogram rarely showed residual mitral regurgitation (*B*). After the XTW system was deployed, mitral regurgitation deteriorated, and aggravation of the jet was observed from the centre and medial sides of the clips (*C*). Openings between the clips were noted, which led to mitral regurgitation deterioration (*D*). LA, left atrium; LV, left ventricle.

Blood tests on admission revealed elevated natriuretic peptide (BNP: 61.2 pg/mL; reference value: ≤18.4 pg/mL). Transthoracic echocardiography revealed severe MR, primarily emanating from the medial side of Segment 2, accompanied by a concentric jet due to the dilated mitral annulus with a flat valve, enlarged left atrium, and reduced left ventricular ejection fraction (LVEF) of 51%. Severe tricuspid regurgitation with non-coated valve leaflets and patent foramen ovale (PFO) were also noted. Transoesophageal echocardiogram showed that the length of the PML was 8.8 mm, and that of the anterior mitral leaflet (AML) was >28.4 mm. A three-dimensional colour Doppler echocardiographic measurement by TEE revealed a moderate-to-severe MR of the vena contracta area of 0.38 cm^2^ with a regurgitant volume of 51 mL (*Figure 1A*, Supplementary material online, *Video S1*). Although his calculated Society of Thoracic Surgeons Predicted Risk of Mortality (STS-PROM) score for mitral valve repair was 5.37%, his clinical frailty scale was 6. Therefore, our team opted for TEER for MR and simultaneous closure of the iatrogenic atrial septal defect (iASD) and PFO.

**Figure 2:**
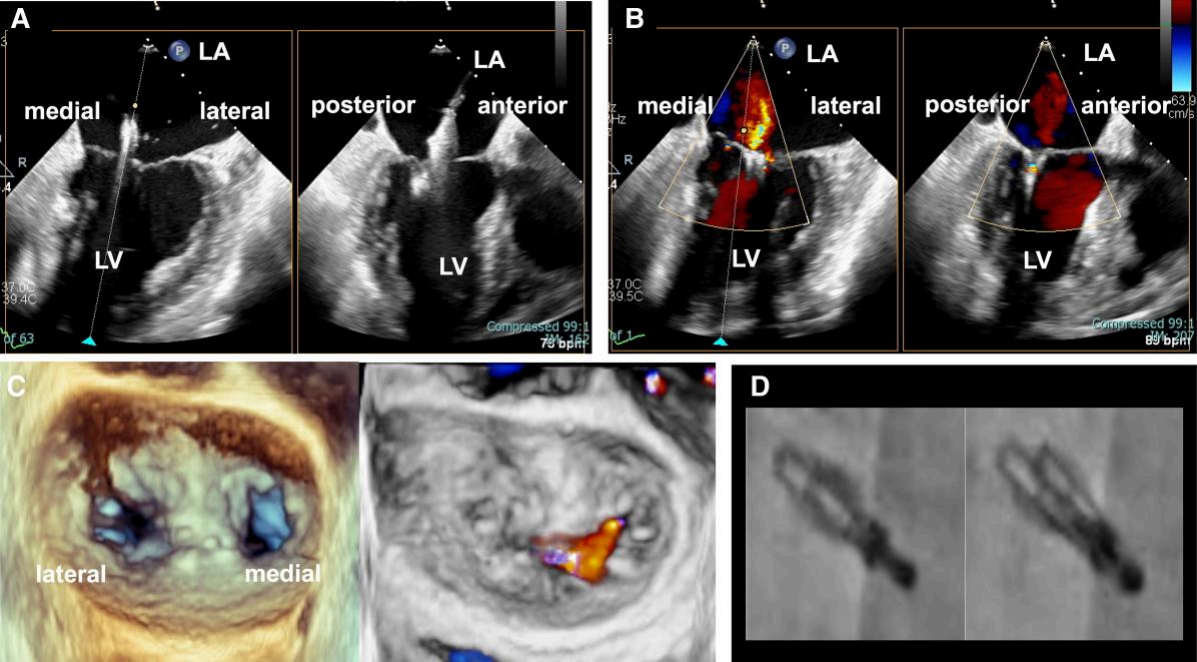
Intra-procedural transoesophageal echocardiogram images of the second transcatheter edge-to-edge mitral valve repair and fluoroscopic images of XTW MitraClip of Patient 1. An additional MitraClip G4 XT system was inserted immediately, medial to the original XTW system (*A*). Mild residual mitral regurgitation was observed immediately after XT system deployment (*B*). The final three-dimensional transoesophageal echocardiogram demonstrated rigid coaptation, and rarely showed residual mitral regurgitation (*C*). Fluoroscopic images of XTW MitraClip just after deployment (*D*, left panel), and openings between the clips were visible (*D*, right panel). LA, left atrium; LV, left ventricle.

The operation was performed under general anaesthesia with TEE guidance. We attempted to grasp the medial side of Segment 2 by using the MitraClip G4 XTW system. The XTW system was aligned perpendicular to the middle of the A2/P2 scallops and subsequently inserted into the left ventricle. The clip was closed, and TEE confirmed adequate leaflet insertion and reduced MR with a transmitral mean pressure gradient of 2 mmHg (*Figure 1B*, Supplementary material online, *Video S2*). However, as soon as the clip was released, MR deteriorated. Precise TEE scanning revealed aggravation of the MR jet from the centre and medial side of the clips. Furthermore, the gaps between the clips were opened (*Figure 1C* and *D*, Supplementary material online, *Video S3*). In addition to TEE, we confirmed COWL using fluoroscopy at right anterior oblique angulation (*Figure 2D*). Therefore, we added a second clip (G4 XT system) adjacent to the medial side of the first clip (*Figure 2A–C*, Supplementary material online, *Video S4*). Subsequently, we implanted a 30 mm GORE® CARDIOFORM ASD Occluder (WL Gore & Associates, Flagstaff, AZ, USA) via iASD, so the PFO could be closed simultaneously. This patient was discharged without any other complications 4 days after TEER. Furthermore, during the follow-up period, the patient remained asymptomatic and showed no signs of heart failure or MR deterioration.

### Patient 2

An 82-year-old woman with a diagnosis of massive MR (regurgitant volume of 65 mL) due to posterior MVP was referred to our institution presenting with shortness of breath. She had a history of TAR with a 26 mm four-branch J Graft SHIELD NEO® (Japan Lifeline Co. Ltd, Shinagawa, Tokyo, Japan) for acute Stanford type A aortic dissection at our institution 2 years prior.

On admission, blood pressure and pulse rate were 97/55 mmHg and 60 b.p.m., respectively. Oxygen saturation was 96% on room air. Physical examination revealed a Levine IV/VI degree pansystolic murmur at apex and pitting leg oedema. His NYHA class was III.

**Figure 3:**
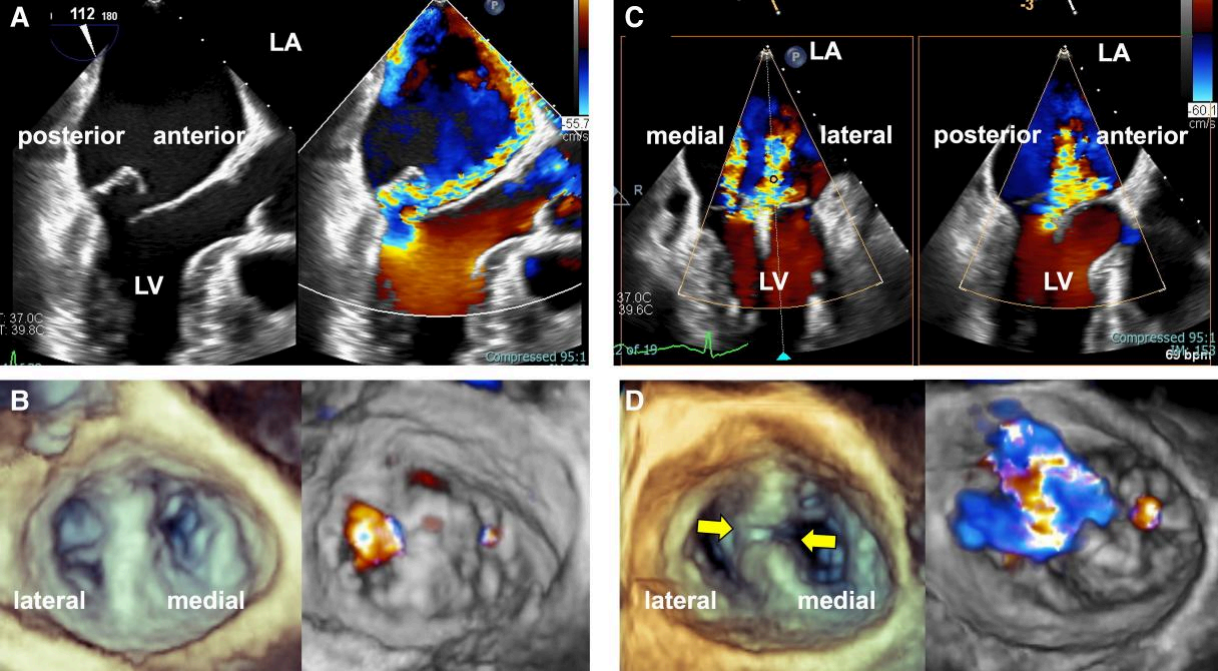
Intra-procedural transoesophageal echocardiogram images of the first transcatheter edge-to-edge mitral valve repair of Patient 2. Preprocedural transoesophageal echocardiogram showing severe mitral regurgitation due to a flailing prolapsed posterior leaflet (*A*). After XTW system deployment, three-dimensional transoesophageal echocardiogram rarely showed residual mitral regurgitation (*B*). Urgent transoesophageal echocardiogram revealed mitral regurgitation deterioration (*C*). An urgent transoesophageal echocardiogram revealed that the implanted XTW clip at Segment 2 was open, forming gaps between the clips and resulting in mitral regurgitation deterioration (*D*). LA, left atrium; LV, left ventricle.

Blood tests on admission revealed elevated natriuretic peptide (BNP: 548.2 pg/mL; reference value: ≤18.4 pg/mL). Transthoracic echocardiography revealed severe MR due to a flail valve in the posterior middle scallop (P2), an enlarged left atrium, and a preserved LVEF of 68%. Transoesophageal echocardiogram measurements showed that the lengths of the PML, AML, and prolapse gap were 15.3, 28.2, and 7.2 mm, respectively, and a flail valve width of 10 mm. A three-dimensional colour Doppler echocardiographic measurement by TEE revealed a large MR of vena contracta area of 0.75 cm^2^ with a regurgitant volume of 107 mL (*Figure 3A*, Supplementary material online, *Video S5*). Although the patient’s STS-PROM score for mitral valve repair was 4.49%, the patient’s general appearance was very weak with a clinical frailty scale of 5. Based on the high surgical risk, our heart team decided to perform TEER for MVP.

The operation was performed under general anaesthesia with TEE guidance. Conventionally, we attempted to grasp Segment 2 using the XTW system; however, due to anatomical malpositioning after TAR surgery, it was difficult to deliver the clip delivery system (CDS). We used the ‘A’ knob to ensure sufficient height and the ‘-’ knob to align the clip that is in reverse aorta hugger. Subsequently, we inserted the clip slightly into the medial side of Segment 2 and grasped them. Transoesophageal echocardiogram confirmed adequate insertion of the leaflets, broad grasping of both scallops, and mild MR with a transmitral mean pressure gradient of 2–3 mmHg (*Figure 3B*, Supplementary material online, *Video S6*).

**Figure 4:**
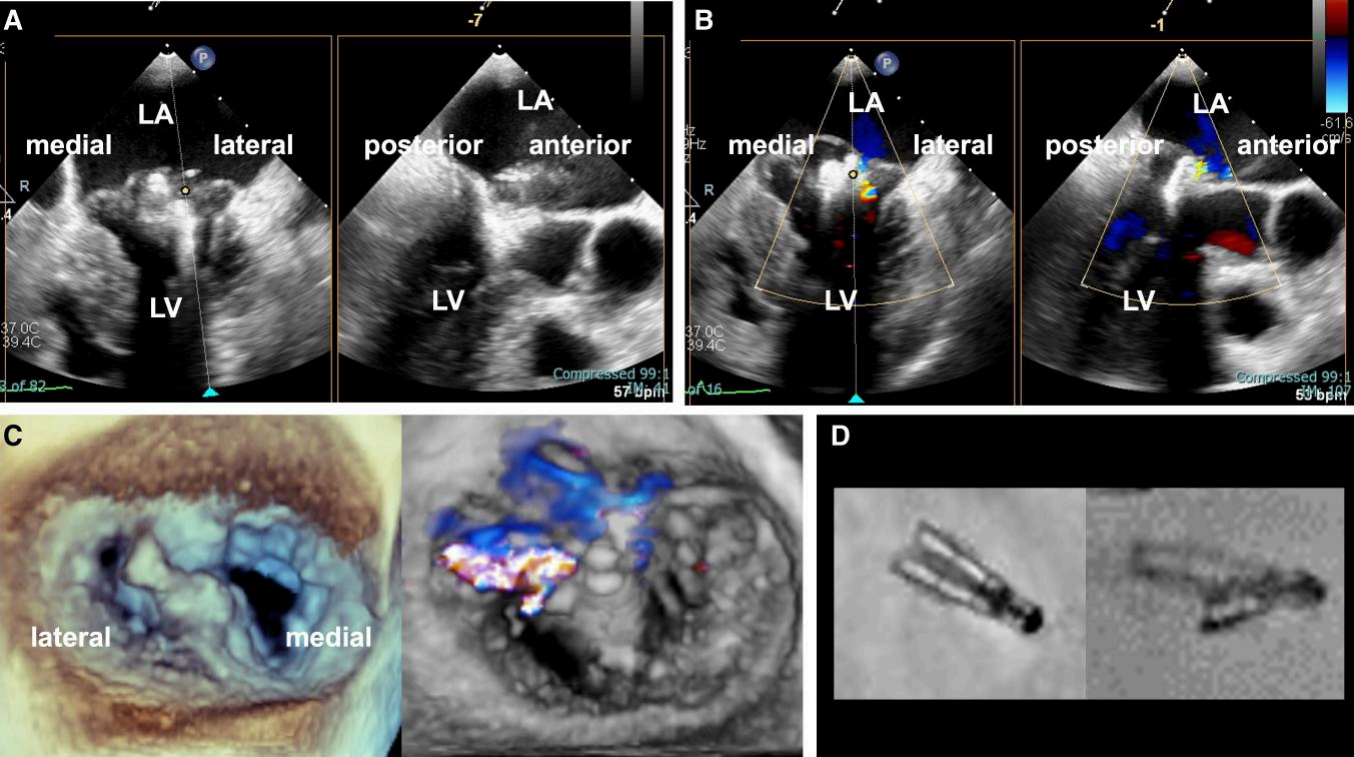
Intra-procedural transoesophageal echocardiogram images of the second transcatheter edge-to-edge mitral valve repair and fluoroscopic images of XTW MitraClip of Patient 2. An additional MitraClip G4 XT system is inserted lateral to the original XTW system (*A*). No residual mitral regurgitation was observed immediately after XT system deployment (*B*). The final three-dimensional transoesophageal echocardiogram demonstrated rigid coaptation, and slight residual mitral regurgitation was observed (*C*). Fluoroscopic images of the XTW MitraClip just after deployment (*D*, left panel), and openings between the clips were visible (*D*, right panel). LA, left atrium; LV, left ventricle.

However, on the second day after TEER, while there were no signs of dyspnoea or leg oedema, an enhanced systolic murmur was notable, and TTE showed worsening of MR. On the same day, we performed TEE, which showed that the implanted XTW clip was opened, forming gaps between the clip, and the MR deteriorated (*Figure 3C* and *D*, Supplementary material online, *Video S7*). Therefore, we performed TEER again to determine the stability of the clip with the COWL on the following day. Intraoperative fluoroscopy confirmed COWL at an anterior–posterior angulation (*Figure 4D*). We successfully inserted an XT clip just lateral to the original XTW clip and reduced the MR (*Figure 4A–C*, Supplementary material online, *Video S8*). This patient was discharged without any other complication 6 days after the second TEER. Furthermore, during the follow-up period, the patient remained asymptomatic and showed no signs of heart failure or MR deterioration.

## Discussion

Here, we report two cases in which COWL occurred at different time points after TEER using the MitraClip G4 system. To the best of our knowledge, there are no reports of COWL after TEER, including those in which COWL caused device embolization. Transcatheter edge-to-edge mitral valve repair with the MitraClip G4 system has been reported to be associated with excellent reduction in MR to ≤2+ in 96.6% of patients at 30 days.^2^ The G4 system has four size variations and can perform grasping with greater flexibility to access complex lesions.^3^

The achievement rate of device success at 30 days, defined as the absence of procedural mortality or stroke, proper placement and positioning of the device, freedom from unplanned surgical or transcatheter interventions related to the device or access procedure, and continued intended safety and performance of the device, was very high (73.0–96.5%), and the low incidence of single leaflet device attachment (SLDA) and device embolization was low (0.0–1.7%).^2,3^ The incidence of COWL after TEER was also low (0.28–0.5%), despite appropriate clip placement with a sufficient decrease in MR (urgent medical device correction report from Abbott, which was released on 8 September 2022, https://www.cardiovascular.abbott/content/dam/cv/cardiovascular/pdf/reports/MitraClip-Urgent-Medical-Device-Correction-September-2022.pdf). Our case series highlights several important facets of rare complications of TEER. One patient showed worsening MR immediately after clip deployment, possibly because of a structural problem with the device itself. The second patient experienced worsening of the MR a few days after treatment with the XTW system, possibly due to the presence of a flail valve.

The mechanism of COWL is presumed to be partially similar to that of SLDA, including excessive mobility, thickening, and calcification of valve leaflets. However, insufficient insertion of leaflet tissue into the clip is the main cause of SLDA, and the frequency of SLDA has decreased with the introduction of the G4 system, increased operator experience, and the establishment of appropriate image assessment methods.^4^ Although operator carelessness or errors and failure to check for adequate clip insertion could also be considered a cause of COWL, we deployed the clip after confirmation of adequate clip insertion in both of our clinical cases. If the clip arm is not perpendicular to the commissure line or if the CDS is not vertical to the horizontal plane of the mitral valve when grasping the valve leaflets, asymmetrical tension on the valve leaflet may occur. After placement, the clip momentarily experiences a rotational force in the direction of rotation due to this asymmetry, which leads to additional stress on the clip. Moreover, these two cases shared a common factor in the complexity of the valvular anatomy. In Patient 1, there may have been high tension on the clip due to taut leaflets and a lack of symmetry in the leaflet insertion due to the flat valve. In Patient 2, difficulties in delivering the CDS to the appropriate position in the left atrium and the presence of a flail leaflet contributed to the overall complexity of the procedure and triggered COWL. In addition, it is necessary to assume that COWL may occur due to problems with the clip device itself, and when MR worsens due to COWL, prompt placement of a second clip should be considered.

## Conclusion

Although the incidence of COWL is low, it can occur during the acute phase following TEER. Clip opening while locked should be considered one of the differential diagnoses when an exacerbation of MR is observed, even immediately after TEER.

## Supplementary Material

ytae322_Supplementary_Data

## Data Availability

Data sharing is not applicable to this article as no data sets were generated or analysed during the current study. The data underlying this article are available in the article and online supplementary material.

## Lead author biography

Hiroki Matsuzoe, MD, PhD, is a cardiologist in Kindai University Faculty of Medicine, Osaka, Japan. He trained in an echocardiography laboratory at Kobe University Graduate School of Medicine from 2014 to 2018 and earned his doctor of philosophy in medicine (PhD) in 2018. He is an expert in echocardiography, valvular heart disease, cardiomyopathy, and heart failure. He was also engaged in interventional cardiology, such as coronary artery disease and vascular disease, and device therapy for cardiac arrhythmias in Yodogawa Christian Hospital from 2018 to 2023. Currently, his interest is in heart failure with structural heart disease, and he is also engaged in clinical research on heart failure.
